# Outcomes of Digital Biomarker–Based Interventions: Protocol for a Systematic Review of Systematic Reviews

**DOI:** 10.2196/28204

**Published:** 2021-11-24

**Authors:** Hossein Motahari-Nezhad, Márta Péntek, László Gulácsi, Zsombor Zrubka

**Affiliations:** 1 Doctoral School of Business and Management Corvinus University of Budapest Budapest Hungary; 2 Health Economics Research Center University Research and Innovation Center Obuda University Budapest Hungary; 3 Corvinus Institute for Advanced Studies Corvinus University of Budapest Budapest Hungary

**Keywords:** digital biomarker, outcome, systematic review, meta-analysis, digital health, mobile health, Grading of Recommendations, Assessment, Development and Evaluation, AMSTAR-2, review, biomarkers, clinical outcome, interventions, wearables, portables, digestables, implants

## Abstract

**Background:**

Digital biomarkers are defined as objective, quantifiable, physiological, and behavioral data that are collected and measured using digital devices such as portables, wearables, implantables, or digestibles. For their widespread adoption in publicly financed health care systems, it is important to understand how their benefits translate into improved patient outcomes, which is essential for demonstrating their value.

**Objective:**

The paper presents the protocol for a systematic review that aims to assess the quality and strength of the evidence reported in systematic reviews regarding the impact of digital biomarkers on clinical outcomes compared to interventions without digital biomarkers.

**Methods:**

A comprehensive search for reviews from 2019 to 2020 will be conducted in PubMed and the Cochrane Library using keywords related to digital biomarkers and a filter for systematic reviews. Original full-text English publications of systematic reviews comparing clinical outcomes of interventions with and without digital biomarkers via meta-analysis will be included. The AMSTAR-2 tool will be used to assess the methodological quality of these reviews. To assess the quality of evidence, we will evaluate the systematic reviews using the Grading of Recommendations, Assessment, Development and Evaluation (GRADE) tool. To detect the possible presence of reporting bias, we will determine whether a protocol was published prior to the start of the studies. A qualitative summary of the results by digital biomarker technology and outcomes will be provided.

**Results:**

This protocol was submitted before data collection. Search, screening, and data extraction will commence in December 2021 in accordance with the published protocol.

**Conclusions:**

Our study will provide a comprehensive summary of the highest level of evidence available on digital biomarker interventions, providing practical guidance for health care providers. Our results will help identify clinical areas in which the use of digital biomarkers has led to favorable clinical outcomes. In addition, our findings will highlight areas of evidence gaps where the clinical benefits of digital biomarkers have not yet been demonstrated.

**International Registered Report Identifier (IRRID):**

PRR1-10.2196/28204

## Introduction

The advent of new medical technologies such as sensors has accelerated the process of collecting patient data for relevant clinical decisions [1], leading to the emergence of a new technology, namely digital biomarkers. By definition, “digital biomarkers are objective, quantifiable, physiological, and behavioral measures collected using digital devices that are portable, wearable, implantable, or digestible” [2].

In addition to their role in routine clinical care, digital biomarkers also play a significant role in clinical trials [3]. Digital biomarkers are considered important enablers of the health care value chain [4], and the global digital biomarker market is projected to grow at a rate of 40.4% between 2019 and 2025, reaching US $5.64 billion in revenue by 2025 [5].

By providing reliable disease-related information [6], digital biomarkers can offer considerable diagnostic and therapeutic value in modern health care systems as monitoring tools and as part of novel therapeutic interventions [7]. Digital biomarkers could reduce clinical errors and improve the accuracy of diagnostic methods for patients and clinicians using measurement-based care [8]. As an alternative to cross-sectional surveillance or prospective follow-ups with a limited number of visits, these technologies can provide more reliable results through continuous and remote home-based observation, and when combined with appropriate interventions, digital biomarkers have the potential to improve therapeutic outcomes [9]. In addition, predicting patients' disease status during continuous monitoring provides opportunities for treatment processes with fewer complications [10].

Given the recent rapid pace of the development of digital health technologies such as software [11], sensors [12], or robotic devices [13,14], their widespread adoption in publicly financed health care systems requires a systematic evaluation and demonstration of their clinical benefits and economic value [15]. The new European Medical Device Regulations effective from May 2021 seek sufficient clinical evidence with the goal of improving clinical security and providing equitable access to appropriate products [16].

Because of rapid technological changes, several potential user groups, and a wide range of functionalities, assessing the value of digital health technologies is a challenging, multidimensional task that often involves broader issues than standard health economic evaluations [17-20]. The National Institute for Clinical Excellence (NICE) has published an evidence framework to guide innovators on what is considered a good level of evidence to support the evaluation of digital health technology. According to the NICE framework, digital biomarkers can fall into several digital health technology risk categories, ranging from simple consumer health monitoring to digital health interventions that potentially impact treatment or diagnosis of care. Although evidence of measurement accuracy and ongoing data collection on use may be sufficient for lower risk categories, for high-risk technologies, demonstration of clinical benefits in high-quality interventional studies is required as a minimum standard of evidence. NICE considers randomized controlled trials (RCTs) conducted in a relevant health care system or meta-analyses of RCTs to be the best practice evidence standard [20].

Numerous studies have conducted systematic reviews of digital biomarkers in recent years with varying results. For instance, a meta-analysis found that implantable cardioverter defibrillators (ICDs) are generally effective in reducing all-cause mortality in patients with nonischemic cardiomyopathy [21], whereas another reported that ICD therapy for the primary prevention of sudden cardiac death in women does not reduce all-cause mortality [16]. In a meta-analysis comparing ICDs with drug treatments, ICDs were found to be more effective than drugs in preventing sudden cardiac death [22]. Some systematic reviews on the use of wearable sensors for monitoring Parkinson disease have reported that wearable sensors are the most effective digital devices to detect differences in standing balance between people with Parkinson disease and control subjects [23] and improve quality of life [24]. In another systematic review, the clinical utility of wearable sensors in patients with Parkinson disease to support clinical decision making was not clear [25]. A 2011 systematic review confirmed no differences between the effectiveness of portable coagulometers and conventional coagulometers in monitoring oral anticoagulation [26].

The inconsistent results from current systematic reviews call for a more systematic assessment of the strength and quality of evidence regarding the health outcomes of interventions based on digital biomarkers. Lack of knowledge about or omission of the quality of evidence of systematic reviews may lead to biased therapeutic guidelines and economic evaluations, and consequently to the widespread adoption of potentially harmful practices and a lag in the adoption of beneficial interventions [27].

Several systems for assessing the quality of evidence have been developed [28], of which the Grading of Recommendations, Assessment, Development and Evaluation (GRADE) system has been adopted by organizations such as the World Health Organization, American College of Physicians, and the Cochrane Collaboration due to its simplicity, methodological rigor, and usefulness in systematic reviews, health technology assessments, and therapeutic guidelines [27]. By assessing study limitations, inconsistency of results, indirectness of evidence, imprecision and reporting bias, GRADE classifies the quality of evidence into four levels from high to very low, with high quality indicating that further research is unlikely to alter our confidence in the effect estimate. Furthermore, by assessing the risk and benefit profile of interventions, GRADE offers two grades of recommendation—strong or weak, with strong recommendations indicating a clear positive or negative balance of risks and benefits [27]. However, some systematic reviews do not provide a structured assessment of the quality of synthesized evidence, and the quality of reporting may also limit the quality assessment of their results. Therefore, the AMSTAR-2 tool was developed as a validated tool to assess the methodological quality of systematic reviews [29].

Our goal is to provide innovators and policy makers with practical insights into the state of evidence generation on digital biomarkers, a rapidly evolving area of medicine [2]. This systematic review of systematic reviews will assess the overall strength of evidence and the reporting quality of systematic reviews that report a quantitative synthesis of the impact of digital biomarkers on health outcomes when compared to interventions without digital biomarkers. Methodological quality of the studies will be assessed using the AMSTAR-2 tool, whereas overall quality of evidence will be evaluated according to GRADE by digital biomarker technologies and reported outcomes.

## Methods

This protocol was prepared following the PRISMA-P (Preferred Reporting Items for Systematic Reviews and Meta-Analyses Protocols) statement preferred for describing items for systematic review and meta-analysis protocols [30]. When reporting the results of this study, amendments or deviations from this protocol will be reported.

### Eligibility Criteria

Original full-text English publications of systematic reviews that report meta-analyses of clinical outcomes of digital biomarker–based interventions compared with alternative interventions without digital biomarkers will be included. Specifically, we will include studies examining digital biomarkers used for diagnosing humans with any health condition in any age group and across genders. Studies investigating the use of digital biomarkers in animals will be excluded. Furthermore, the definition of digital biomarkers may overlap with sensor applications in the general population such as citizen sensing [31]. In this research, we will only consider systematic reviews focusing on digital devices used by clinicians or patients with the aim of collecting clinical data during treatment.

All interventions that involve the use of digital biomarkers for any purpose related to diagnosing patients, monitoring outcomes, or influencing the delivery of a therapeutic intervention will be considered. There will be no restrictions on comparators as long as the comparator arm does not involve the application of digital biomarkers for the purposes listed above. Only meta-analyses of clinical outcomes that report the intentional or unintentional change in the health status of participants resulting from an intervention will be considered. Systematic reviews that focus on measurement properties or other technical or use-related features of digital biomarkers that are not measures of a change in participants' health status due to an intervention are not eligible for this review.

Systematic reviews published between January 1, 2019, and December 31, 2020, will be included. We will include full-text articles published in English in peer-reviewed journals, conference papers, or systematic review databases, as well as full-text documents of systematic reviews from non–peer-reviewed sources, such as book chapters or health technology assessment reports.

### Search Strategy

A comprehensive literature search will be conducted in PubMed and the Cochrane Library. In addition, the reference list of eligible full-text systematic reviews will be searched for other potentially eligible reviews for our study. Keywords related to “digital biomarkers” and filters for “systematic reviews” and publication dates will be combined in the literature search. Automatic expansion of the search terms to include applicable MeSH (Medical Subject Headings) terms will be allowed. For searching the digital biomarker studies, we operationalized the definition of “digital biomarkers” [2]. For identifying systematic reviews, the search filter proposed by the National Library for Medicine will be used [32]. This filter was designed to retrieve systematic reviews from PubMed that have been assigned the publication type “Systematic Review” during MEDLINE indexing, citations that have not yet completed MEDLINE indexing, and non-MEDLINE citations. The full syntax is provided in Table 1. An equivalent syntax will be developed to retrieve Cochrane reviews from the Cochrane Library.

**Table 1:** Search expressions for PubMed.

Terms	Number	Syntax
Digital biomarkers	#1	“digital biomarker” OR “digital biomarkers” OR portable OR portables OR wearable OR wearables OR implantable OR implantables OR digestible OR digestibles
Systematic reviews	#2	(((systematic review[ti] OR systematic literature review[ti] OR systematic scoping review[ti] OR systematic narrative review[ti] OR systematic qualitative review[ti] OR systematic evidence review[ti] OR systematic quantitative review[ti] OR systematic meta-review[ti] OR systematic critical review[ti] OR systematic mixed studies review[ti] OR systematic mapping review[ti] OR systematic Cochrane review[ti] OR systematic search and review[ti] OR systematic integrative review[ti]) NOT comment[pt] NOT (protocol[ti] OR protocols[ti])) NOT MEDLINE [subset]) OR (Cochrane Database Syst Rev[ta] AND review[pt]) OR systematic review[pt]
Publication date	#3	(“2019/01/01”[Date - Publication]: “2020/12/31”[Date - Publication])
Final search strategy	#4	#1 AND #2 AND #3

### Screening and Selection

After removing duplicates, 2 reviewers will independently screen the titles and abstracts according to two main eligibility criteria: (1) systematic reviews and (2) interventions including digital biomarkers that meet the definition “objective, quantifiable, physiological, and behavioral measures collected using digital devices that are portable, wearable, implantable, or digestible” [2]*.* Following this definition, imaging or any other technology that does not measure physiological or behavioral data will be excluded from this study. Portable, wearable, implantable, or digestible medical devices or sensors, which generate physiological or behavioral data, will be considered as digital biomarkers (such as fitness trackers and defibrillators). We interpret portable as “portable with respect to patients or consumers”; therefore, portable devices that are operated by health care professionals (eg, digital stethoscopes) will be excluded. Studies other than systematic reviews will be excluded in the screening phase. Interreviewer calibration exercises will be performed after title and abstract screening of the first 100 records, using the following method: both screening criteria will be scored as 1 if “criterium not met” and 0 if “criterium met or unsure.” Therefore, reviewers can evaluate each record by assigning a score of 1, 2, 3, or 4, denoting response patterns of (0,0), (1,0), (0,1), and (1,1), respectively. Interrater agreement and the κ statistic will be calculated for the score, and reviewers will be retrained if worse than substantial agreement (κ 0.6) is not observed [33]. In case of discordant evaluations, a third reviewer will make decisions.

After screening, full-text articles will be evaluated against all eligibility criteria by 2 independent reviewers: (1) is the language English? (yes/no or unsure), (2) does the review concern human studies? (yes/no or unsure), (3) was the review published between January 1, 2019, and December 31, 2020? (yes/no or unsure), (4) does the review involve a meta-analysis of clinical outcomes? (yes/no or unsure), (5) does the intervention involve a digital biomarker used for diagnosis, patient monitoring, or influencing therapy? (yes/no or unsure), (6) does the comparator arm lack a digital biomarker for the same purposes? (yes/no or unsure). For inclusion, all 6 criteria must have yes as the answer. Discrepancies will have to be resolved by the 2 reviewers. In case of disagreement, a third reviewer will make the decision on including the article. Excluded full-text articles and the reasons for exclusion will be included as an appendix to the publication of results.

**Figure 1:**
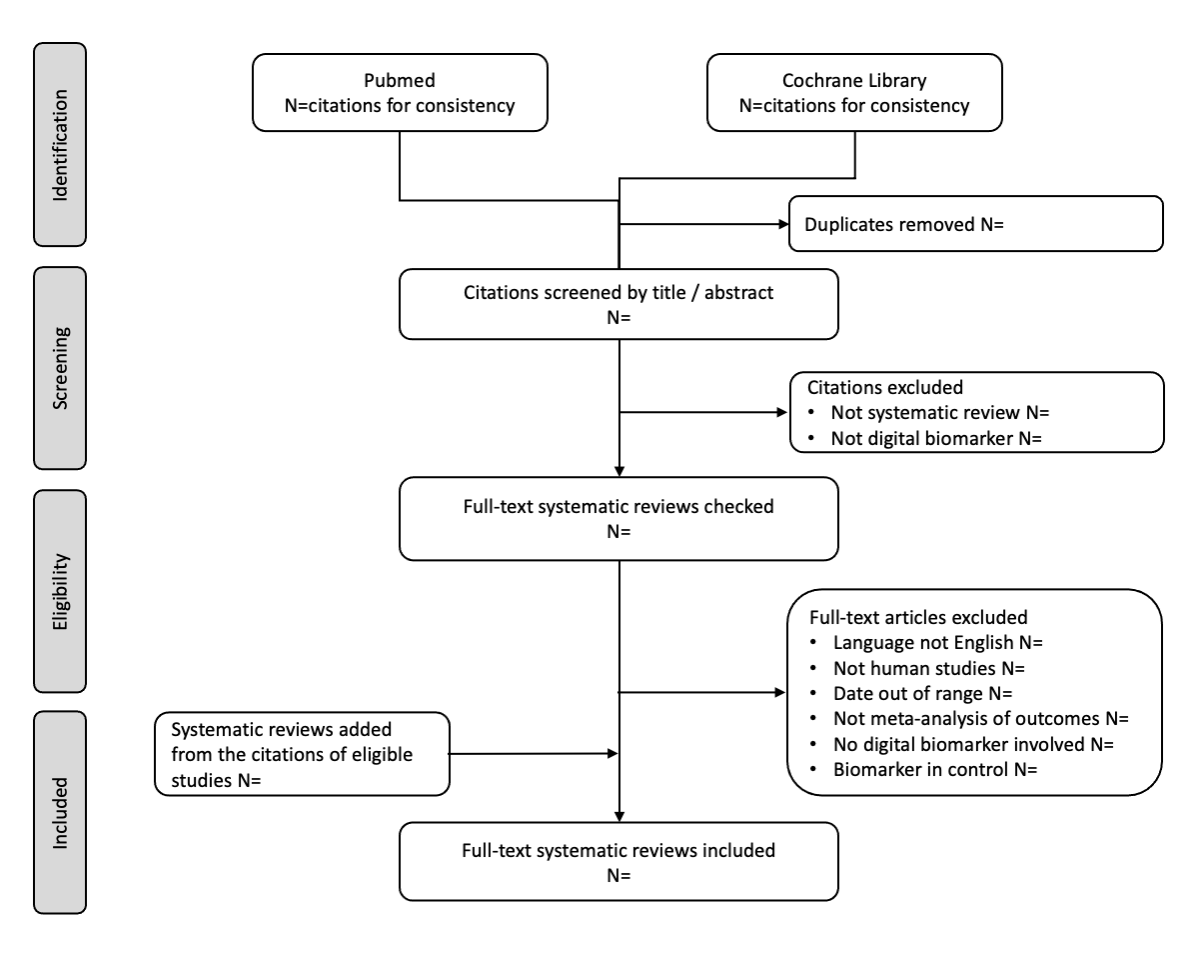
PRISMA-P (Preferred Reporting Items for Systematic Reviews and Meta-Analyses Protocols) flow diagram of included studies.

The screening results and selection of eligible studies will be visualized using the PRISMA-P 2009 flow diagram shown in Figure 1 [34].

### Data Extraction

Data extraction will be performed by 2 independent researchers, and a calibration exercise (evaluation of interrater agreement) will be conducted after completing data extraction from 20% of the included studies. Discrepancies between reviewers will be resolved by consensus, and residual differences will be settled by a third reviewer. Any modification needed in the data extraction form will be done at this point.

### Study-Level Variables

**Figure 2:**
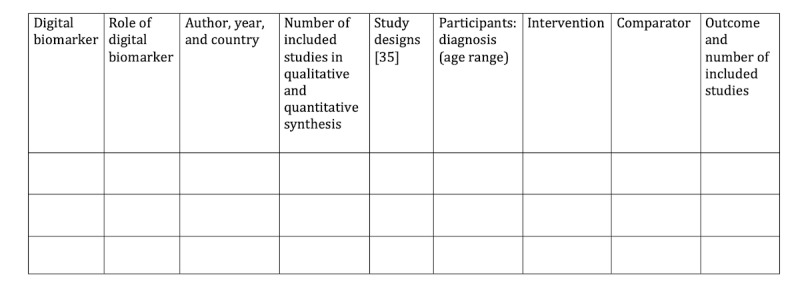
Summary of the included studies, which will include resources retrieved from non–peer-reviewed sources and reviews retrieved from peer-reviewed sources. Study designs will be listed in abbreviated form as the following: randomized controlled trial (RCT), non–randomized controlled trial (non-RCT), cohort study (C), case-control study (CC), and cross-sectional study (CS).

We will record the following study-level variables: year of publication, the first author’s country using code 3166-1 of the International Standards Organization, the total number of included studies on qualitative and quantitative synthesis as well as separately for every outcome, study designs of the included studies (RCTs and non-RCTs, cohort studies, case-control studies, and cross-sectional studies) [35], population and its age range, disease condition using the International Classification of Diseases 11th Revision coding [36], intervention, type of intervention using the International Classification of Health Interventions coding [37], comparator, type of comparator, digital biomarker, role of digital biomarker (diagnosis, patient monitoring, and influencing intervention), bodily function quantified by the digital biomarker using the International Classification of Functioning, Disability and Health coding [38], and the list of synthesized outcomes. Each eligible study will be summarized in Figure 2.

### Assessment of the Methodological Quality of Systematic Reviews

The methodological quality of the eligible systematic reviews will be assessed using the criteria of the AMSTAR-2 tool [29] by 2 independent reviewers. Discrepancies will be resolved by consensus; lingering differences will be resolved by a third reviewer. AMSTAR-2 is a reliable and valid tool used for assessing the methodological quality of systematic reviews of randomized and nonrandomized studies of health care interventions [29,39]. In brief, AMSTAR-2 evaluates methodological quality according to the following 16 criteria: (1) research question according to the PICO (patient, intervention, comparison, outcome) framework, (2) methods established prior to the study, (3) explicit inclusion criteria, (4) comprehensive literature search, (5) study selection in duplicate, (6) data extraction in duplicate, (7) reporting of excluded studies, (8) detailed description of included studies, (9) risk of bias (RoB) assessment, (10) disclosure of funding sources, (11) appropriate statistical methods for evidence synthesis, (12) quantitative assessment of RoB in main results, (13) study-level discussion of RoB, (14) explanation for heterogeneity of results, (15) investigation of publication bias, and (16) reporting conflicts of interest.

**Figure 3:**
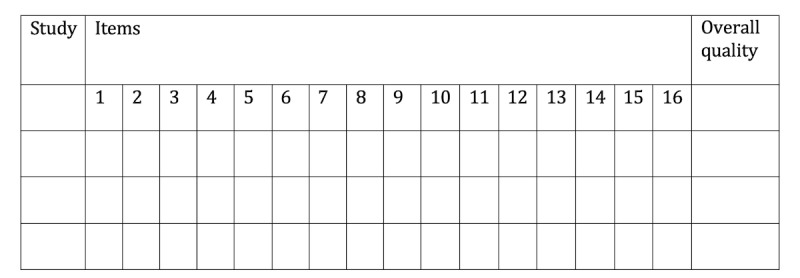
Assessment of the methodological quality of reviews (AMSTAR-2). Overall quality will be listed as critically low (CL), low (L), medium (M), and high (H).

For consistent rating [40], we will use the AMSTAR-2 website [41]. The AMSTAR-2 website provides an overall grading of the studies in four categories: critically low, low, medium, and high. It also provides explicit criteria for the answer options (yes, partially yes, and no). For each eligible article, answers for all AMSTAR-2 items and the overall ratings will be presented in Figure 3.The AMSTAR-2 items are presented in Textbox 1.

**AMSTAR-2 items.**

1. Did the research questions and inclusion criteria for the review include the components of the PICO (patient, intervention, comparison, outcome) framework?
2. Did the review report contain an explicit statement that the review methods were established prior to conducting the review and did the report justify any significant deviations from the protocol?
3. Did the review authors explain their selection of the study designs for inclusion in the review?
4. Did the review authors use a comprehensive literature search strategy?
5. Did the review authors perform study selection in duplicate?
6. Did the review authors perform data extraction in duplicate?
7. Did the review authors provide a list of excluded studies and justify the exclusions?
8. Did the review authors describe the included studies in adequate detail?
9. Did the review authors use a satisfactory technique for assessing the risk of bias (RoB) in individual studies that were included in the review?
10. Did the review authors report on the sources of funding for the studies included in the review?
11. If a meta-analysis was performed, did the review authors use appropriate methods for the statistical combination of results?
12. If a meta-analysis was performed, did the review authors assess the potential impact of RoB in individual studies on the results of the meta-analysis or other evidence synthesis?
13. Did the review authors account for RoB in individual studies when interpreting and discussing the results of the review?
14. Did the review authors provide a satisfactory explanation for and discussion of any heterogeneity observed in the results of the review?
15. If they performed a quantitative synthesis, did the review authors carry out an adequate investigation of publication bias (small study bias) and discuss its likely impact on the results of the review?
16. Did the review authors report any potential sources of conflicts of interest, including any funding they received for conducting the review?

### Outcome-Level Variables

**Figure 4:**
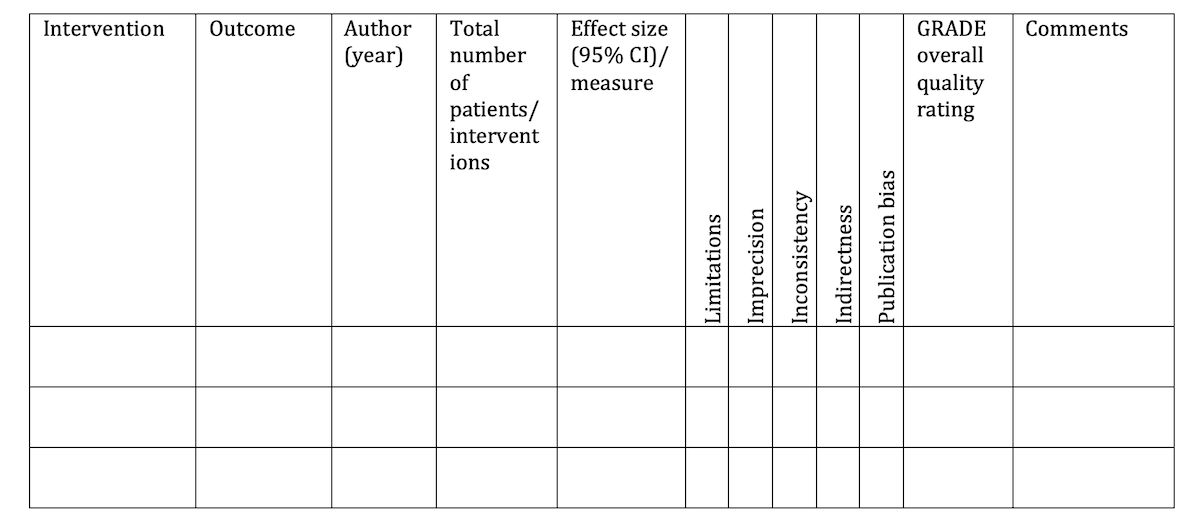
Evidence summary and quality assessment by the GRADE (Grading of Recommendations, Assessment, Development and Evaluation) tool. Measure will be listed as risk ratio (RR), odds ratio (OR), mean difference (MD), and standardized mean difference (SMD). GRADE certainty ratings will be provided as high (H), medium (M), low (L), and very low quality of evidence (VL).

In addition to study-level variables, for each outcome synthesized in the meta-analyses, the following information will be extracted by duplicate reviews, using the process described above: the measured outcome, total number of studies per outcome, total number of patients and number of patients in the intervention, effect size and its 95% CI (upper and lower limits), as well as the type of effect size (standardized mean difference, odds ratio, and risk ratio). Quantitative descriptions of outcomes will be grouped by digital biomarker and are provided in Figure 4, along with the assessment of the quality of evidence.

### Assessing the Quality of Evidence

We will evaluate the quality of evidence of the meta-analyses for each outcome using the GRADE system [27,42]. By default, GRADE considers the evidence from RCTs as high quality; however, this assessment may be downgraded for each outcome based on the evaluation of the following quality domains: (1) RoB [43], (2) inconsistency [44], (3) imprecision [45], (4) publication bias [46], and (5) indirectness [47]. Depending on the severity of the quality concerns, for each domain, a downgrade of 0, 1, or 2 can be proposed. We will assign downgrades for RoB according to the following criteria: if 75% or more than 75% of the included studies for a given outcome are reported to have low RoB, no downgrades will be assigned; if less than 75% of the included studies have low RoB, 1 downgrade will be assigned; if the RoB is not reported, 1 downgrade will be allocated [48].

To evaluate inconsistency, the reported heterogeneity (*I*^2^ statistic) of studies for each outcome will be considered. If the heterogeneity of the included studies of an outcome is less than or equal to 75%, no downgrade will be allotted. If the heterogeneity of included studies of the outcome is more than 75%, 1 downgrade will be assigned. If only 1 trial is included in the outcome, no downgrade will be assigned. In cases where the heterogeneity is not reported, we will assign 1 downgrade [48].

To assess imprecision, the sample size and CI will be evaluated [49]. Following the broad recommendations of the GRADE handbook [42], we will apply no downgrade if the pooled sample size is over 2000. We will apply 1 downgrade if the pooled sample size is less than 200. For pooled sample sizes between 200 and 2000, we will assess the optimal information size criterion as follows [42]: by expecting a weak effect size of 0.2 [50], we will calculate the sample size for an RCT using the pooled standard error and pooled sample size assuming a balanced sample, power of 0.8, and significance level of .05. If the calculated sample size is greater than the pooled sample size, 1 downgrade will be applied [42,49].

Publication bias appears when a pooled estimate does not comprise all the studies that could be included in the evidence synthesis [51]. One way to detect publication bias is to visually observe a funnel plot. Owing to the limitations of the funnel plot [46,52], this method may not show publication bias accurately [49,53] and may lead to false conclusions [52,54]. Therefore, we will assess publication bias using the trim and fill method proposed by Duval and Tweedie [55]. Potentially missing studies will be imputed, and the pooled effect size of the complete data set will be recalculated. In case the imputation of potentially missing studies change the conclusions of the analysis (eg, a significant effect size will not be significant anymore), we will apply 1 downgrade attributable to publication bias [55].

When assessing indirectness, any differences between the population, interventions, and comparators in each outcome of the research questions of the reviews will be considered [52]. In this regard, the studies included in each meta-analysis outcome will be evaluated. If the population, interventions, or comparators are consistent with the main aims of the meta-analysis, no downgrading will be considered. If the population, interventions, or comparators of the studies do not match the main objectives of the meta-analysis, depending on the severity of this mismatch, a downgrade of 1 or 2 will be considered based on the consensus of the 2 independent researchers involved in data extraction.

The quality evaluation and assignment of downgrades in each domain will be performed by 2 independent reviewers. Discrepancies will be resolved by consensus, and if required, decisions will be made by a third reviewer. The overall grading of the quality of evidence for each outcome will be performed by consensus. As a starting point for the consensus on overall evaluation, we will use the recommendations by Pollock et al [48]: (1) high quality indicates that further research is very unlikely to change our confidence in the effect estimate (0 downgrades); (2) moderate quality means further research is likely to have an important impact on our confidence in the effect estimate and may change the estimate (1-2 downgrades); (3) low quality implies further research is very likely to have an important impact on our confidence in the effect estimate and is likely to change the estimate (3-4 downgrades); (4) very low quality means any effect estimate is very uncertain (5-6 downgrades) [27,48].

In addition to the quantitative description of outcomes, the number of downgrades (0, 1, or 2) for each domain and the overall quality assessment (high, moderate, low, or very low) of the evidence with reasons for downgrades will be presented in Figure 4 for each outcome by each digital biomarker.

If the required information from the eligible studies is lacking at any stage of the research, or in case of ambiguity, we will contact the corresponding authors of the reviews by email to obtain the required information or to remove the ambiguity. If we do not receive any response, the case will be considered as “missing” or “not reported.”

### Evidence Synthesis

Interrater agreement during screening will be evaluated via the percentage of agreement and the Cohen κ statistic. Study characteristics will be summarized using descriptive statistics. Given the heterogeneity of the included populations and interventions, we plan to provide a qualitative synthesis of the results for each digital biomarker by the type of intervention and outcome.

## Results

This protocol was submitted before data collection. Search, screening, and data extraction will commence in December 2021 in accordance with the published protocol. The study is funded by the National Research, Development and Innovation Fund of Hungary (reference number: NKFIH-869-10/2019).

## Discussion

Our study will provide a comprehensive summary of the breadth and quality of evidence available on the clinical outcomes of interventions involving digital biomarkers.

### Strengths

Most of the systematic review studies conducted in the field of digital biomarkers in recent years have been mainly performed with a specific focus on one or more disease areas or technologies such as the effects of wearable fitness trackers on motivation and physical activity [56] or ICD troubleshooting in patients with left ventricular assist devices [57]. To the best of our knowledge, no comprehensive systematic review of systematic reviews has been published on all types of digital biomarkers in all populations and diseases. Therefore, our review aims to assess the quality of methods and evidence of systematic reviews, without being limited to a specific area or technology, using validated tools and standard methodologies. As a result, the strength of evidence can be compared between different types of interventions, providing practical guidance for clinicians and policy makers.

### Limitations

One of the potential limitations of this study is the restricted search time period (2019 and 2020). Owing to the breadth of the scope, we chose a shorter timeframe for our review. However, we hypothesized that considering the new European Medical Device Regulations that were published in 2017 [16], this is a highly relevant period for evaluating the available clinical evidence generated prior to the implementation of the regulations. Furthermore, we hypothesized that given the rapid development of the field [3], systematic reviews are published regularly to summarize key developments in the generation of clinical evidence.

We operationalized the definition of digital biomarkers in our search. However, the sensitivity and specificity of our search filter to retrieve articles concerning digital biomarkers has not been tested. In addition to the general keywords applied in our search expressions, digital biomarkers may be identified by specific terms referring to the technology or type of data collected [3]. However, the creation of a comprehensive list of relevant search terms for all existing technologies was beyond the scope of this study and remains a research question to be answered. Furthermore, we will apply the definition of digital biomarkers in a clinical setting. Some sensor applications in the general population may have public health implications (eg, COVID-19 contact tracing apps [58]) , which will be omitted from this review. The challenges of interpreting the digital biomarker definition will be discussed.

Although relevant guidelines for systematic reviews of systematic reviews recommend searching in the Database of Abstracts of Reviews of Effectiveness (DARE) in addition to PubMed and Cochrane [59], we will limit our search to PubMed and Cochrane when retrieving reviews. It should be noted that the DARE was not used in this study as it does not contain new reviews from 2015.

### Conclusions

In conclusion, our results will help identify clinical areas where the use of digital biomarkers has led to favorable clinical outcomes. Furthermore, our results will highlight areas with evidence gaps where the clinical usage of digital biomarkers has not yet been studied.

## Abbreviations

DARE: Database of Abstracts of Reviews of Effectiveness
GRADE: Grading of Recommendations, Assessment, Development and Evaluation
ICD: implantable cardioverter defibrillator
MeSH: Medical Subject Headings
NICE: National Institute for Clinical Excellence
PICO: patient, intervention, comparison, outcome
PRISMA-P: Preferred Reporting Items for Systematic Reviews and Meta-Analyses Protocols
RCT: randomized clinical trial
RoB: risk of bias
